# COVID-19 associated pulmonary aspergillosis in critically-ill patients

**DOI:** 10.1186/s13613-024-01324-z

**Published:** 2024-07-01

**Authors:** Guangting Zeng, Yuchi Zhou

**Affiliations:** 1 Department of Pharmacy, The First People’s Hospital of Chenzhou, Xiangnan University, Chenzhou, China; 2 Urology Department, Fourth People’s Hospital of Chenzhou, Chenzhou, China

Dear editor,

We read with great interest the multicenter study conducted by Bay et al. that detailed the incidence and outcomes of COVID-19-associated pulmonary aspergillosis (CAPA) in critically-ill patients [1].They reported a low incidence of CAPA(5.1%) in a large multicenter cohort.Although CAPA was not signifcantly associated with day-28 mortality, patients with CAPA experienced a longer duration of mechanical ventilation and ICU stay.We applaud the authors for their work, but we sincerely point out some information errors in the tables, such as the proportion of females in CAPA patients being 17% instead of 28%.

It is well known that the diagnosis of invasive pulmonary aspergillosis remains challenging. Recent studies have shown that the incidence of CAPA disease ranges from 2.4–34.3% [2]. This difference is partly related to the heterogeneous population. But more important is related to screening strategies. In this study, CAPA diagnostic tests were initiated by the attending physicians. However, screening strategies are not well described, whether routine screening, such as routine testing of serum and bronchoalveolar-lavage fluid, is implemented, and what proportion of the population is tested. This information was necessary to understand the design of the study. In addition, whether patient enrollment was performed strictly according to inclusion and exclusion criteria and continuous, as each center appeared to enroll less than 1 patient per month based on the number of patients and centers enrolled and the duration of the study.

Invasive aspergillosis is associated with a poor prognosis in patients with COVID-19.This study did not observe a significant association of CAPA with 28-day mortality, but the mortality rate was still as high as 34%. On the one hand, the small number of people diagnosed with CAPA may not achieve statistical power. Another aspect may be the effect of the widespread use of antifungal agents. Twenty-eight patients (97%) received antifungal therapy during the ICU stay, which seems to be a good signal that antifungal agents are effective in reducing mortality in some patients. Moreover, we were interested in what proportion of patients received antifungal prophylaxis and how this affected survival outcomes. Early identification of high-risk patient phenotypes, timely implementation of antifungal prophylaxis and treatment, and how long the treatment duration are still the directions that need to be explored in the future.

## Data Availability

Our manuscript has no associated data.
